# EEG cortical activity and connectivity correlates of early sympathetic response during cold pressor test

**DOI:** 10.1038/s41598-023-27480-z

**Published:** 2023-01-24

**Authors:** Gianluca Rho, Alejandro Luis Callara, Giulio Bernardi, Enzo Pasquale Scilingo, Alberto Greco

**Affiliations:** 1 Dipartimento di Ingegneria dell’Informazione, University of Pisa, Via G. Caruso 16, 56122 Pisa, Italy; 2 Research Center “E. Piaggio”, University of Pisa, Largo Lucio Lazzarino 1, 56122 Pisa, Italy; 3 MoMiLab Research Unit, IMT School for Advanced Studies Lucca, 55100 Lucca, Italy

**Keywords:** Neuroscience, Biomedical engineering

## Abstract

Previous studies have identified several brain regions involved in the sympathetic response and its integration with pain, cognition, emotions and memory processes. However, little is known about how such regions dynamically interact during a sympathetic activation task. In this study, we analyzed EEG activity and effective connectivity during a cold pressor test (CPT). A source localization analysis identified a network of common active sources including the right precuneus (r-PCu), right and left precentral gyri (r-PCG, l-PCG), left premotor cortex (l-PMC) and left anterior cingulate cortex (l-ACC). We comprehensively analyzed the network dynamics by estimating power variation and causal interactions among the network regions through the direct directed transfer function (dDTF). A connectivity pattern dominated by interactions in $\alpha$ (8–12) Hz band was observed in the resting state, with r-PCu acting as the main hub of information flow. After the CPT onset, we observed an abrupt suppression of such $\alpha$-band interactions, followed by a partial recovery towards the end of the task. On the other hand, an increase of $\delta$-band (1–4) Hz interactions characterized the first part of CPT task. These results provide novel information on the brain dynamics induced by sympathetic stimuli. Our findings suggest that the observed suppression of $\alpha$ and rise of $\delta$ dynamical interactions could reflect non-pain-specific arousal and attention-related response linked to stimulus’ salience.

## Introduction

The autonomic nervous system (ANS) coordinates bodily functions to ensure protective stability for the tissues and the organs (i.e., homeostasis)^1,2^. Such mechanism is not under voluntary control, but it is governed by the synergistic contribution of the sympathetic (SNS) and the parasympathetic (PNS) nervous systems^1^. At the central brain level, these systems are controlled by the central autonomic network (CAN), an intricate network of reciprocally connected areas that extends throughout the entire neural axis^3,4^. One of the nodes of the CAN is represented by the brainstem, where the responses to different kind of external and internal stimuli (e.g., thermal, biomechanical and biochemical) are generated and projected back to the periphery and the higher brain structures (i.e., cortex)^2,3,5^. In the latter case, autonomic responses are integrated with higher-order processes^2,5^, and a top-down modulation of autonomic reflexes based on the integration of painful, cognitive, emotional, sensory and endocrine related information is performed^2,3^.

Under resting-state conditions, PNS regulation dominates over SNS, promoting a decrease of cardio-respiratory rate and dilation of blood vessels^1^. Conversely, in the presence of any physical or mental stressor, complex interactions occur at both central and peripheral level, and generally result in an enhanced response of the SNS and a reduction of PNS activity^2,6^. However, different sympathetic responses may be elicited based on the specific stressor type, as well as other environmental factors^4^. Accordingly, studying the correlates of SNS activation under controlled scenarios should help to better understand the mechanisms underlying autonomic regulation.

A variety of experimental procedures have been devised to study the sympathetic activation in response to different types of stressors, ranging from autonomic tasks such as maximum forced respiration, Valsava maneuver and handgrip tasks, to more complex designs including decision-making and emotional paradigms^3^. Another common sympathetic task is the cold pressor test (CPT), i.e., the immersion of the hand in cold water, which has been shown to elicit one of the most strong and prolonged sympathetic responses^7–9^. The effect of such response has been widely investigated through the analysis of peripheral autonomic correlates. In particular, features extracted from electrodermal activity (EDA) have been shown to reliably reflect the increase in sympathetic activity elicited by the CPT^10–13^. Similarly, studies analyzing the heart rate variability (HRV) have shown an increase in the heart rate and a decrease in the parasympathetic activity during CPT compared to resting-state^13–15^. Furthermore, invasive recordings of muscle sympathetic nerve activity (MSNA) highlighted an effect of CPT sympathetic activation on baroreceptor modulation and peripheral hormonal levels concentration^16,17^.

Since the bodily response to sympathetic arousal involves also changes in brain activity, other studies focused on the central correlates of CPT through functional neuroimaging. The majority of these studies reported a significant increase in neural activity of brainstem nuclei, cerebellum and subcortical structures such as thalamus, amigdala and hippocampus during CPT^18–23^. Some of these changes were further observed to negatively correlate with the heart rate during the CPT^21^. However, brain activity in response to CPT was found also in several cortical regions involved in the processing of pain, memory and emotion regulation, such as the anterior cingulate cortex (ACC), superior frontal gyrus (SFG), inferior frontal gyrus (IFG), insular cortex (INS) and precentral gyrus (PCG)^21,23–25^. Particularly, the activity of both INS and ACC was found to negatively correlate with the heart rate variation during CPT, suggesting the possible implication of these regions in higher-level regulation of baroreflex arc^21,25^. Moreover, the ACC activity was also found to be related to the distraction from pain induced by CPT^24^. Accordingly, investigating brain activity at the cortical level may provide a window on the higher-order processes modulating the CPT sympathetic response. Nevertheless, the activity of significant regions alone may only give a partial view of the processes underlying the CPT, as physiological responses are also the result of how information is integrated among such areas^26^. Therefore, investigating the cortical connectivity can provide novel insights into the regulatory mechanisms underlying the sympathetic response elicited during tasks such as the CPT.

Electroencephalography (EEG) analysis can provide an estimate of cortical electrical activity, allowing to investigate the neural correlates of a plethora of different tasks with an unmatched temporal resolution. Among these, quantitative EEG studies on CPT assessed how EEG power varied, at the channel level, between CPT and resting-state conditions^27–30^. Specifically, during the CPT power increased in both the $\delta$(1–4 Hz) and $\theta$(4–8 Hz) bands at the frontal sites, along with an increase of $\beta$ band(16–30 Hz) power at the temporal and posterior sites^27,28^. Moreover, the power in the $\gamma$ band(30-45 Hz) was significantly higher at the frontal and posterior sites^28^. Conversely, power decreased in the $\alpha$(8–12 Hz) band at the parietal and occipital sites^27–30^. Other studies applied source localization methods to investigate the cortical generators of CPT^30,31^. In particular, during the CPT, activity decreased in the $\alpha$ band in the cingulate cortex, pre- and postcentral gyral regions. Conversely, both $\delta$ and $\theta$ band activities increased in the cingulate, frontal, temporal and insular cortices^31^. Significant increases of cortical activity have also been observed in both the $\beta$ and $\gamma$ bands, although less specific and more widespread^30,31^. Previous studies also applied brain connectivity measures to the EEG channels to classify the perceived level of pain during CPT^32^. In this context, integrating well-established measures of brain activity, such as EEG power at both channel and source level, with more sophisticated techniques such as those provided by effective connectivity analysis (e.g., Granger-causality-derived measures) may highlight significant frequency-specific interactions between active regions and identify a key cortical network of the sympathetic response.

In this work, we investigated the EEG correlates of the sympathetic activation elicited during the CPT through the estimation of both EEG power and connectivity in a group of 26 healthy subjects. Specifically, we evaluated significant differences in the power of EEG channels between the CPT and an initial resting-state condition. Subsequently, we exploited a K-means clustering procedure to identify a common network of active sources at the group-level based on the similarity of subject-specific component scalp maps, and we estimated their dynamic changes in power. Moreover, we estimated time-varying causal interactions before and during the CPT among the network’s brain sources by means of MVAR modeling of their associated time-courses and subsequent direct directed transfer function (dDTF) estimation^33^. We confined both the power and connectivity analysis to the 60 s window ranging from the last 30 s of the initial resting-state and the first 30 s of CPT. This choice was motivated by previous work on these data, in which an early increase of sympathetic arousal within the first 30 s of CPT was proven by EDA and HRV analysis^10,11^.

## Methods

### Subjects

The experimental protocol was approved by the“Comitato Etico Regionale per la Sperimentazione Clinica della Regione Toscana”, section “Area Vasta Nord Ovest” - Protocol n. 7803, Registry number 1072, approved on 18 Jan 2018. The recordings were carried out in agreement with the Declaration of Helsinki. All participants gave their informed consent before the experiment.

Twenty-six healthy volunteers (age 26 ± 3, 8 females, all right-handed) participated in the study. Subjects did not have any history of neurological and cardiovascular diseases, or alcoholic or smoking habits. To exclude subjects affected by mental disorders from the study, volunteers underwent the Patient Health Questionnaire (PHQ)^34^. All volunteers successfully passed the test (PHQ score less than 5), showing no signs of mental disorders.

### Experimental protocol

Participants were asked to sit on a comfortable chair and to keep their eyes closed for the entire duration of the experiment. The experimental protocol consisted of 3 minutes of Cold Pressor Test (CPT) task, preceded by 4 min of initial resting-state and followed by 3 min of final resting-state. During the CPT, participants were manually guided by the experimenter to submerge their non-dominant hand up to the wrist into a basin filled with a mixture of ice and water at the temperature of 0 degrees. The CPT duration was chosen according to previous experiments assessing the average resistance to pain over time in healthy subjects^16^. Participants who could not tolerate the pain for the entire duration of the task were allowed to move to the subsequent final resting-state phase.

### EEG acquisition and preprocessing

EEG data was acquired using a 128-channel Geodesic EEG System 300 from Electrical Geodesic Inc. (EGI). Electrodes were referenced to Cz and grounded through two additional channels placed between Cz and Pz. We kept the electrodes’ impedance always below 20$k\Omega$. We used a sampling frequency of 500 Hz.

EEG was analyzed with the support of EEGLAB^35^ and Fieldtrip^36^. First, we applied a zero-phase low-pass filter (cut-off frequency = 45 Hz, transition band = 10 Hz) and downsampled the data to the sampling frequency of 100 Hz. Then, we created two different datasets, by applying two zero-phase high-pass filters at the cutoff frequencies of 0.1 Hz and 2 Hz, respectively. The 2 Hz-filtered dataset was used to estimate the independent components weights from the EEG signal, whereas the 0.1 Hz-filtered dataset was used to estimate both power and connectivity measures. This choice was mainly due to poor ICA decomposition of the 0.1Hz-filtered dataset. Indeed, slow drift components such as those below 1 Hz can significantly reduce data stationarity, deteriorating the unmixing quality of ICA^37,38^. Accordingly, the 2Hz-filtered dataset was preprocessed by identifying and removing flat channels and poorly correlated (i.e., $\rho <0.80$) channels. Then, high-amplitude and short-time artifacts were corrected by applying the Artifact Subspace Reconstruction (ASR) algorithm^39^. This method performs a spatial filtering on the principal components’ (PCs) space of the data, comparing the variance of each PC with specific thresholds. More in detail, portions of clean EEG data (i.e., the calibration data) are selected based on the distribution of signal’s variance, using a sliding window. The calibration data is then projected to its PC space, and PC-specific thresholds are estimated based on their variance, weighted by a user-defined cut-off parameter. Using the same sliding window approach, the ASR performs a PCA decomposition on the uncleaned portions of EEG data, and those PCs whose variance exceeds the thresholds are removed. The data is finally reconstructed based on the remaining components^27,39^. Increasing the cut-off parameter increases the threshold values and leads to very conservative filtering. On the other hand, decreasing the cut-off parameter decreases the threshold values and leads to very sensitive filtering, causing not only the rejection of artifacts but also the loss of relevant information from the data^40^. Here, we adopted a conservative cut-off parameter of 30, which allows for the rejection of the majority of artifacts while preserving the relevant information in the data^40^. We visually inspected the data to further remove bad data periods not successfully repaired by the ASR^41^. Then, we recovered all the previously removed channels through spherical interpolation and we average-referenced the data. Finally, we decomposed the data into a set of maximally independent components with respect to time, through the Adaptive Mixture Independent Component Analysis (AMICA) algorithm^42^. In particular, this algorithm was observed to reach higher mutual information reduction, while unveiling a greater number of dipolar brain components^43^. Finally, the obtained ICA weights were applied to the 0.1 Hz-filtered dataset after removing and interpolating the same channels excluded during the preprocessing of the 2 Hz-filtered dataset.

Eight subjects were discarded from further analyses due to poor EEG signal quality, resulting in data from 18 subjects (age 27±4, 8 females). Moreover, we excluded from the analyses the time interval between −5 s and +5 s around the start of the CPT (i.e., 0 s), due to the presence of strong muscular artifacts induced by the immediate cold sensation and the physical interference introduced by the experimenter guiding the subjects’ hand into the water.

### Network identification

We used the ICLabel EEGLAB plug-in to identify components related to brain activity or to artefacts. In particular, ICLabel tagged ICs as *brain, eye, muscle, heart, line-noise, channel-noise* and *other*^44^. Here, based on such classification, we removed all the components labeled as non-brain. Then, we visually inspected the remaining ICs to eventually remove misclassified artefact-related components. Afterwards, brain ICs of each subject were grouped together and clustered with a K-means algorithm whose feature vector was made of the first 10 PCs of each component scalp map. We identified the optimal number of clusters as the minimum of the Davies-Bouldin index^45^ in the (2, 20) range. Finally, we ran the K-means with 500 replicates for reliability reasons.

For each subject, ICs assigned to the same cluster were averaged together. Then, the scalp map of each cluster centroid was described by means of an equivalent current dipole. Specifically, dipoles were located within a Boundary Element Model (BEM) of the head based on the Montreal Neurological Institute (MNI) template through a two-step procedure implemented in Fieldtrip^36^. First, a coarse localization on a three-dimensional grid was obtained. Such grid consisted of 34 equally spaced points ranging from −85 mm to + 85 mm along both the X- and Y-axis, and 17 equally spaced points ranging from 0mm to +85mm along the Z-axis. The X, Y, Z axes extended left-right, posterior-anterior, and inferior-superior respectively, and the axes origin coincided with the MNI space origin. Then, a nonlinear optimization of dipoles’ position was performed based on the minimization of the difference between the the scalp map described by the dipole and the observed scalp potential. On this network, we performed the subsequent power and connectivity analyses.

### Power spectral density analysis

We first reconstructed the EEG signal of each subject from the only contribution of its clustered ICs activity. Then, we estimated the power spectral density (PSD) for each EEG channel within the time intervals from −35 s to −5 s (i.e., resting-state) and from + 5 s to + 35 s (i.e., CPT task) with respect to the CPT start (i.e., 0 s). To compute the PSD we applied the Welch’s method with 5 s-long moving windows with 80$\%$ of overlap.

In addition, for each clustered IC (i.e., brain source), we estimated a time-varying PSD using a sliding time window of 5 s and time-step of 1 s, within the time range from +5 s to +35 s with respect to the CPT start (i.e., 0 s). Each time step has been then compared with the PSD estimated in the last available 5s of resting-state (i.e., from −10 s to −5 s) (see section "Statistical analysis").

For both analyses, PSD estimates were then averaged in the $\delta$, $\theta$, $\alpha$, $\sigma$, $\beta$ and $\gamma$ bands.

### Connectivity analysis

We estimated causal interactions among the ICs of the obtained network in the range from −35 s to −5 s and from +5 s to +35 s with respect to the CPT start. In particular, we exploited the instantaneous temporal independence among components to limit zero-lag issues affecting GC-based measures estimated on the channel space, as well as to enhance the interpretability of ICs in terms of brain regions contributing to the network^46^. Accordingly, we implemented the following MVAR model:

1 $$X(t) = \sum\limits_{{k = 1}}^{p} {A(k)X(t - k) + \varepsilon (t)} {\text{ }}$$

where $X\in \Re ^{N \times M}$ are the time courses of the network’s ICs, *N* is the number of ICs and M is the length of the time series. In particular, at each time *t*, the time series $X (t)$ were modeled as a linear combination of their past values from $t-1$ to $t-p$, where *p* is the order of the MVAR model, weighted by coefficients of the matrix $A(k)\in \Re ^{N \times N}$, plus an error term $\epsilon (t)$. Each coefficient $a_{ij}(k)$ quantifies the contribution of the j-th time series at time $t-k$ to the current value of the i-th time series, whereas the error term is assumed as white Gaussian with covariance matrix $\Sigma$.

We estimated MVAR models through the Vieira-Morph algorithm, using a moving window of 5 s and an overlap of 4 s. Specifically, for each subject we estimated the optimal model order in the range (1–20) according to four different information criteria (i.e., AIC, BIC, FPE, HQ). Then, we fitted MVAR models with $p=p_{avg}=7$ as the average over the optimal model orders across the subjects. We validated the models by testing their consistency, stability and whiteness of residuals^47,48^. Finally, we estimated direct causal interactions among the network ICs through the dDTF in the (1–45) Hz frequency range^33^:

2 $$\begin{aligned} dDTF_{ij}(f) = \eta _{ij}(f) \chi _{ij}(f) \end{aligned}$$

where $\eta _{ij}$ is the full-frequency Directed Transfer Function (ffDTF):

3 $$\begin{aligned} \eta _{ij}(f)^2 = \frac{|H_{ij}(f)|^2}{\sum _{f=f_{min}}^{f_{max}}\sum _{m=1}^{N}|H_{im}(f)|^2} \end{aligned}$$

and $\chi _{ij}$ is the Partial Coherence (PCoh):

4 $$\begin{aligned} \chi _{ij}(f)^2 = \frac{P_{ij}(f)^2}{P_{ii}(f)P_{jj}(f)} \end{aligned}$$

Briefly, the ffDTF is obtained from the transfer matrix *H*(*f*) of the MVAR model:

5 $$\begin{aligned} H(f)=(I-A(f))^{-1} \end{aligned}$$

where *A*(*f*) is the Fourier transform of the coefficients matrix *A*(*k*), and gives a measure of directional causal interactions between the network nodes. On the other hand, PCoh (4) exploits the inverse spectral matrix $P(f) = S(f)^{-1} = (H(f) \Sigma H^*(f))^{-1}$ to quantify the direct coupling between two nodes (i.e., pathways which does not involve intermediate nodes). As result, the combination of the properties of ffDTF with those offered by PCoh endows the dDTF of the capability of estimating directed causal interactions between time series^33^.

### Statistical analysis

In this section, we describe the three statistical analyses performed to evaluate the cortical activity and connectivity significantly associated with the CPT. First, we compared the PSD at the channel level between the CPT and the resting-state conditions. Secondly, we compared the time-varying PSD of each clustered ICs estimated during the CPT with the PSD estimated over the last available 5s of the resting state. Thirdly, we statistically tested for non-zero causal interactions among clustered ICs. More in detail:

1. for each frequency band and each EEG channel, we tested for significant differences between the PSD estimated during the CPT and the resting-state with a Montecarlo-permutation procedure with 10000 permutations, followed by a cluster correction procedure to handle multiple hypothesis testing^49^;
2. for each frequency band and for each clustered IC, we tested for significant differences between the PSD evaluated during the last 5s of resting-state and the PSD evaluated over consecutive 5s-long windows, using a non-parametric Wilcoxon signrank test for each comparison ($p<0.05$); we controlled for multiple comparisons using the false discovery rate (FDR) procedure for mutiple hypothesis testing under dependency^50^;
3. we assessed the significance of causal interactions through surrogate data testing with a phase randomization approach^51^. More specifically, we computed 5000 surrogates to obtain the distribution of the dDTF representing the null-hypothesis of no causal interaction for each combination of (*i*, *j*, *f*, *t*). Notably, the dDTF distribution is estimated on surrogate time series that hold the same amplitude properties of the original time-series but whose phase information has been randomly assigned in range $(0,2\pi )$^51^. We performed a group-level analysis by comparing the median dDTF(*i*, *j*, *f*, *t*) value across subjects with the median surrogate distribution for each connection (*i*, *j*), frequency (*f*) and time-window (*t*). dDTF values whose associated *p*-value was $<0.05$ were considered significant. Multiple testing was controlled with FDR^50^.

### Ethical approval

The experimental protocol was approved by the “Comitato Etico Regionale per la Sperimentazione Clinica della Regione Toscana”, section “Area Vasta Nord Ovest” - Protocol n. 7803, Registry number 1072, approved on 18 Jan 2018. The recordings were carried out in agreement with the Declaration of Helsinki. All participants gave their informed consent before the experiment.

## Results

### Network identification

We identified an average of 81 ICs across subjects, for a number of 1455 ICs in total. After removing ICs not related to brain activity based on their ICLabel classification, their associated scalp map, power spectrum and time-course, we obtained an average of 22 ICs per subject.

**Figure 1:**
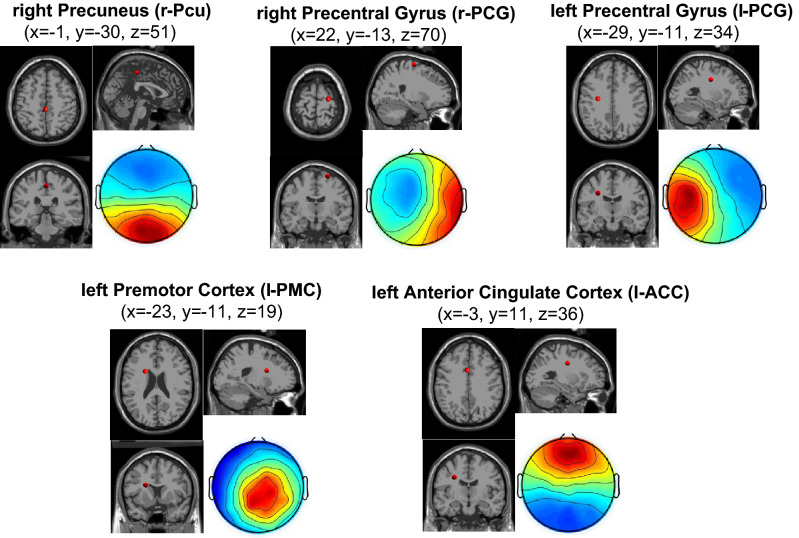
Results of K-means clustering of brain ICs across subjects. The scalp map related to each centroid is obtained as the average of single-subject scalp maps assigned to the cluster. Associated equivalent current dipoles are depicted in red within a standard MRI template and labeled according to the nearest portion of gray matter.

The DB index score, evaluated on the outcome of K-means for a number of cluster centroids in the range from 2 to 20, showed a minimum (i.e., the optimum value) using 5 centroids. Accordingly, we clustered the ICs into 5 clusters. In Fig. 1 we report the centroids of the network along with their associated equivalent current dipoles.

Specifically, for each node of the network we report the scalp map as well as the dipole position projected on the axial, sagittal and coronal planes, respectively. We labeled network nodes based on the nearest region of gray matter to the *x*, *y*, *z* position of each source dipole in the MNI space. Accordingly, we assigned network nodes in the right Precuneus (r-Pcu; MNI coordinates $x=-1, y=-30, z=51$), right Precentral Gyrus (r-PCG; MNI coordinates $x=22, y=-13, z=70$), left Precentral Gyrus (l-PCG; MNI coordinates $x=-29, y=-11, z=34$), left Premotor Cortex (l-PMC; MNI coordinates $x=-23, y=-11, z=19$), and left Anterior Cingulate Cortex (l-ACC; MNI coordinates $x=-3, y=11, z=36$).

### Power spectral density analysis

**Figure 2:**
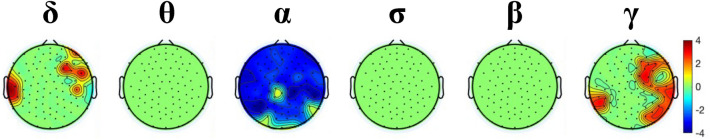
Statistical comparison of channels’ power spectral density (PSD) between resting-state and cold pressor test (CPT) in the $\delta$(1–4 Hz), $\theta$(4–8 Hz), $\alpha$(8–12 Hz), $\sigma$(12–16 Hz), $\beta$(16–30 Hz) and $\gamma$(30–45 Hz) bands, conducted with a Student t-test ($p<0.05$, corrected with cluster-correction). Each scalp map represent the distribution of t-values for which the comparison was significant. Positive t-values indicate higher power during CPT, with respect to resting-state, whereas negative t-values indicate lower power during CPT, with respect to resting-state.

In Fig. 2, we report the results of the PSD changes for each scalp channel between the initial resting-state (i.e., from − 35 s to − 5 s) and CPT (i.e., from + 5 s to + 35 s) conditions, for the $\delta$, $\theta$, $\alpha$, $\sigma$, $\beta$ and $\gamma$ bands. We observed a significant increase of power in the $\delta$ and $\gamma$ bands during CPT, with respect to the rest. Specifically, $\delta$-PSD was higher in the left-temporal and right frontal areas. Similarly, $\gamma$-PSD was higher in the left-temporal area and in the right hemisphere, although differences were more widespread for this band. On the other hand, we observed a decrease of PSD during CPT, with respect to the resting-state, in the $\alpha$ band. Such variation involved almost all the electrodes, with the exception of few differences in the left-central, occipital and right-temporal areas that were not significant. We did not observe any significant difference between conditions for the $\theta$, $\sigma$ and $\beta$ bands.

**Figure 3:**
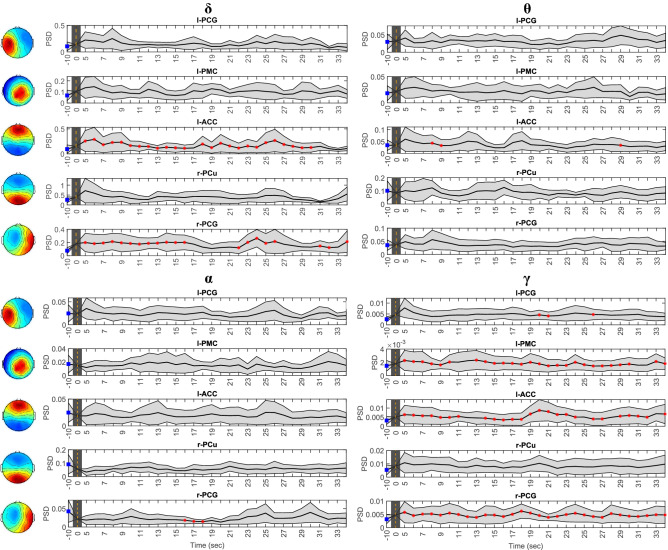
Median ± MAD of network nodes’ power spectral density (PSD) in the last 5 s window of resting-state (i.e., from − 10 s to − 5 s; "*Blue square*") and in the first 30 s of cold pressor test (CPT) (i.e., from +5 s to +35 s), for the $\delta$, $\theta$, $\alpha$, and $\gamma$ bands. The CPT start is marked by a dashed line at 0s. Each time point represent the value of PSD evaluated within a 5 s-long window, with 1 s shift. Significant differences ("*Red asterisk*") between consecutive time widows of CPT and the last 5 s of resting-state were assessed with a Wilcoxon signrank test ($p<0.05$, FDR-corrected). We report only those frequency bands for which we observed significant differences.

Figure 3 depicts the results of the statistical comparison between the PSD evaluated over the last 5 s of the initial resting-state (i.e., from −10 s to −5 s) and the time-varying PSD evaluated at each time step of 1 sec. We considered the first 30 s of the CPT session (i.e., from + 5 s to + 35 s).

In particular, we only report the results for those frequency bands in which we observed significant differences. We found a significant increase of power in the $\delta$ band for both the l-ACC and r-PCG regions. More specifically, the power of l-ACC was higher from the start to nearly the end of CPT, whereas the power of r-PCG was higher for about the first 10 s of the task. We also found an increase of power for the l-ACC within the first seconds of CPT and again towards the end of the task in the $\theta$ band. On the other hand, we observed a decrease of power in the $\alpha$ band for the r-PCG after about 12 s from the start of CPT. Finally, we observed a significant increase of power in the $\gamma$ band for the l-PCG, l-PMC, l-ACC, and r-PCG. Specifically, the power of l-PCG was significant at about 15 s after the start of CPT, whereas the power of l-PMC, l-ACC, and r-PCG was higher for nearly the whole observed interval of the task.

### Connectivity analysis

**Figure 4:**
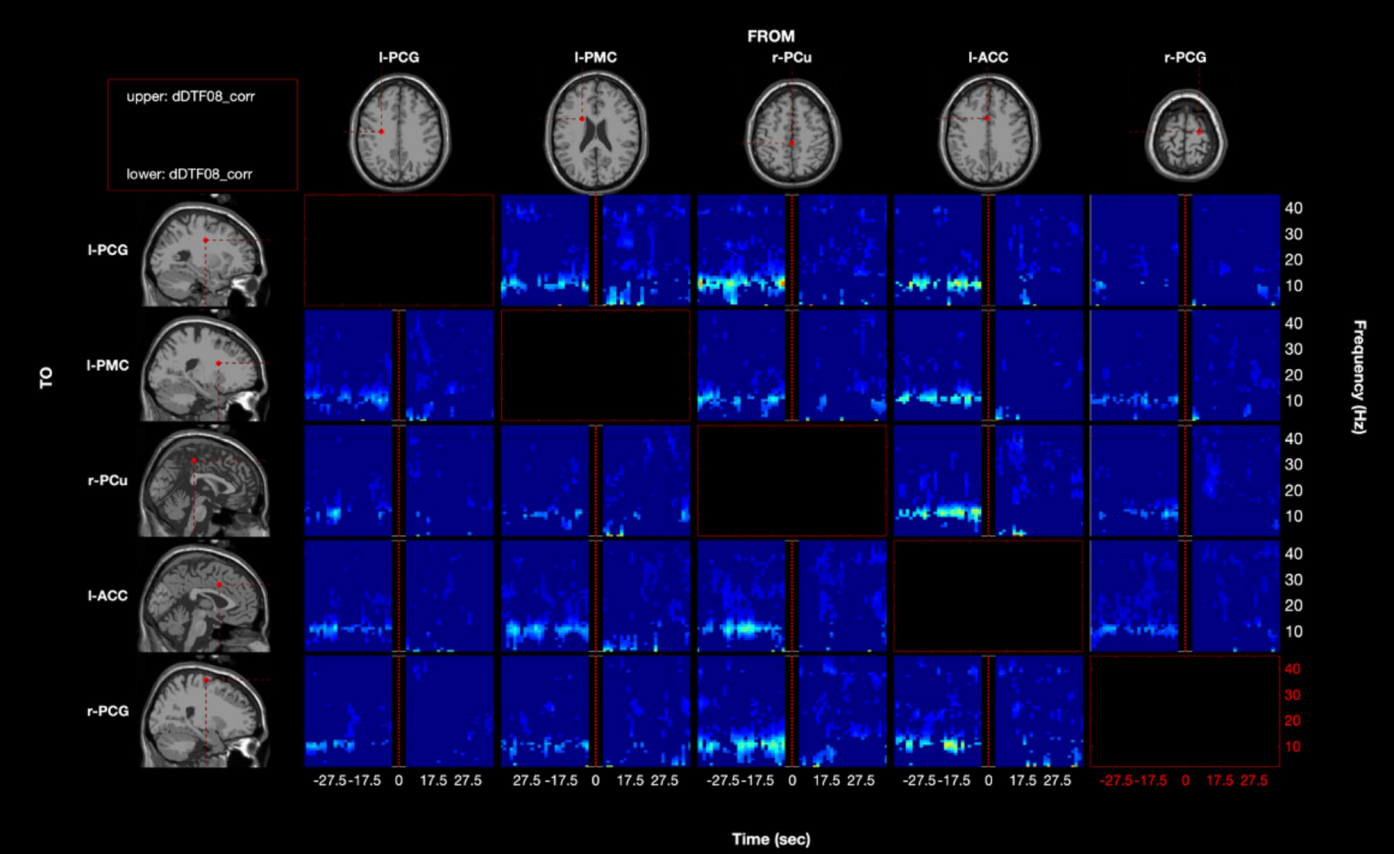
Time-frequency representation of group-median direct Directed Transfer Function (dDTF) between network nodes (i.e., l-PCG, l-PMC, r-PCu, l-ACC and r-PCG). Only values significantly different from 0 after surrogate data testing with phase randomization are reported ($p<0.05$, corrected with FDR). Time is represented along the x-axis, whereas frequency is represented along the y-axis. Intersections between columns and rows depict directional causal information flow from one node (i.e., source) to another (i.e., sink). dDTF was evaluated in the time range from − 35 s to − 5 s (i.e., resting-state) and from +5s to +35s (i.e., CPT), with respect to the CPT start (i.e., vertical red dashed line at 0s), using a 5s moving window with a shift of 1s, and in the frequency range from 1Hz to 45Hz. Each point over time accounts for the dDTF between − 2.5 s and − 2.5 s. We did not analyze windows overlapping between resting-state and CPT (i.e., black areas from − 5 s to + 5 s).

Figure 4 depicts the results of connectivity analysis between the network nodes at the group-level.

Specifically, we report the time-frequency representation of dDTF median values across subjects for each combination of sources *j* (i.e., columns) and sinks *i* (i.e., rows), in the 1–45 Hz frequency range and in the time intervals from − 35 s to − 5 s and + 5 s to + 35 s around the CPT start. Only dDTF values which significantly differed from 0 ($p<0.05$, corrected with FDR) are shown.

**Figure 5:**
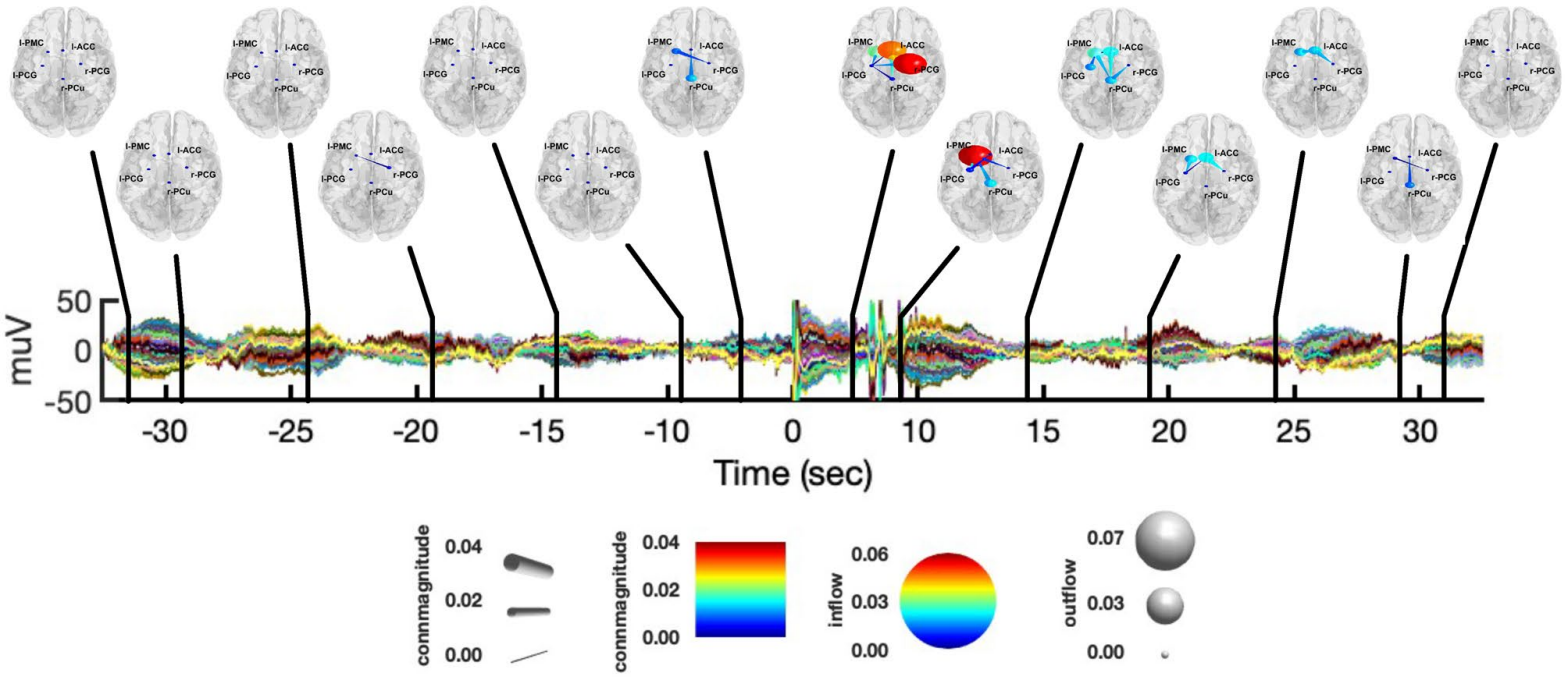
Graphical representation of causal interactions between the network nodes (i.e., l-PCG, l-PMC, r-PCu, l-ACC and r-PCG) integrated in the $\delta$ band, during both the resting-state from − 35 s to − 5 s and CPT from + 5 to + 35, with respect to the CPT start. Nodes are reported in a standard template, with their corresponding label. Node size represent information outflow, whereas node color represents information inflow. The edges represent connections between nodes, whose color and size encode connectivity magnitude. The directionality of connections is given by the direction were the edges taper.

In Fig. 5, we report the observed causalities integrated in the $\delta$ band, at different time instants, during both the initial resting-state and CPT. During the CPT, l-PMC was the node with the biggest outflow, sending information to all the other nodes during the first seconds of stimulation. More specifically, while l-PMC$\rightarrow$r-PCG was significant only for the first windows of CPT, we observed information outflows towards r-PCu, l-PCG, l-ACC for about 10 s from the start of the task. Additionally, we found a late information flow from l-PMC to r-PCG at around + 27.5 s after the stimulation onset. Similar to l-PMC, the l-ACC showed significant amounts of causal outflows during the entire observation interval of CPT. More precisely, within the first two windows of stimulation we observed both l-ACC$\rightarrow$l-PMC and l-ACC$\rightarrow$r-PCu to be significant, with l-ACC$\rightarrow$r-PCu being significant again at around + 12.5 s. Moreover, we observed late causal l-ACC outflows to l-PCG at about 15.5 s, and from l-ACC to r-PCG towards the end of the stimulation interval. From the start of CPT, we also observed causal inflows to l-PMC from both r-PCG and l-PCG, as well as significant interactions from r-PCu to r-PCG. Concerning the resting-state, we did not observe any specific pattern of causal interactions.

**Figure 6:**
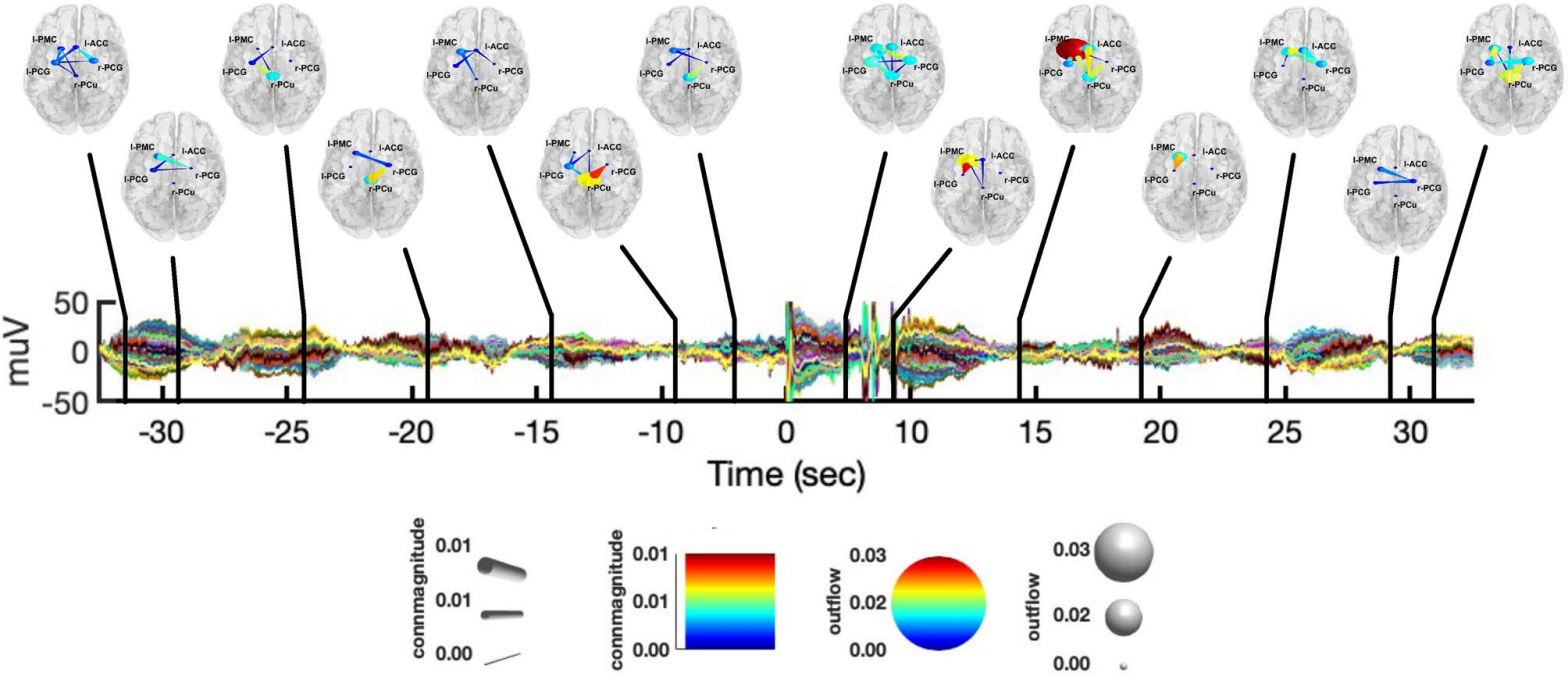
Graphical representation of causal interactions between the network nodes (i.e., l-PCG, l-PMC, r-PCu, l-ACC and r-PCG) integrated in the $\theta$ band, during both the resting-state from − 35 to − 5 s and CPT from + 5 to + 35 s, with respect to the CPT start. Nodes are reported in a standard template, with their corresponding label. Node size represent information outflow, whereas node color represents information inflow. The edges represent connections between nodes, whose color and size encode connectivity magnitude. The directionality of connections is given by the direction were the edges taper.

In Fig. 6 we report the causal interactions between network nodes integrated in the $\theta$ band, during both the resting-state and CPT and at different points over time.

During CPT we found causal interactions similar to those observed in the $\delta$ band, whereas causal interactions were less specific and more widespread during the resting-state. From the start of CPT, we found causal outflows from l-PMC to both l-PCG, r-PCu and l-ACC. More precisely, both l-PMC$\rightarrow$l-PCG and l-PMC$\rightarrow$l-ACC were found to be significant up to 10s after CPT start. Similarly, for the first windows of CPT, l-PMC also received information inflows from l-PCG, r-PCG and l-ACC. Furthermore, l-PMC received inflows from r-PCu at about 12.5s and 22.5 s after the CPT start. In the last windows of CPT, we observed causal interactions from r-PCG to l-PCG, r-PCu and l-ACC. On the other hand, we observed a causal inflow to r-PCG from both l-ACC and r-PCu.

**Figure 7:**
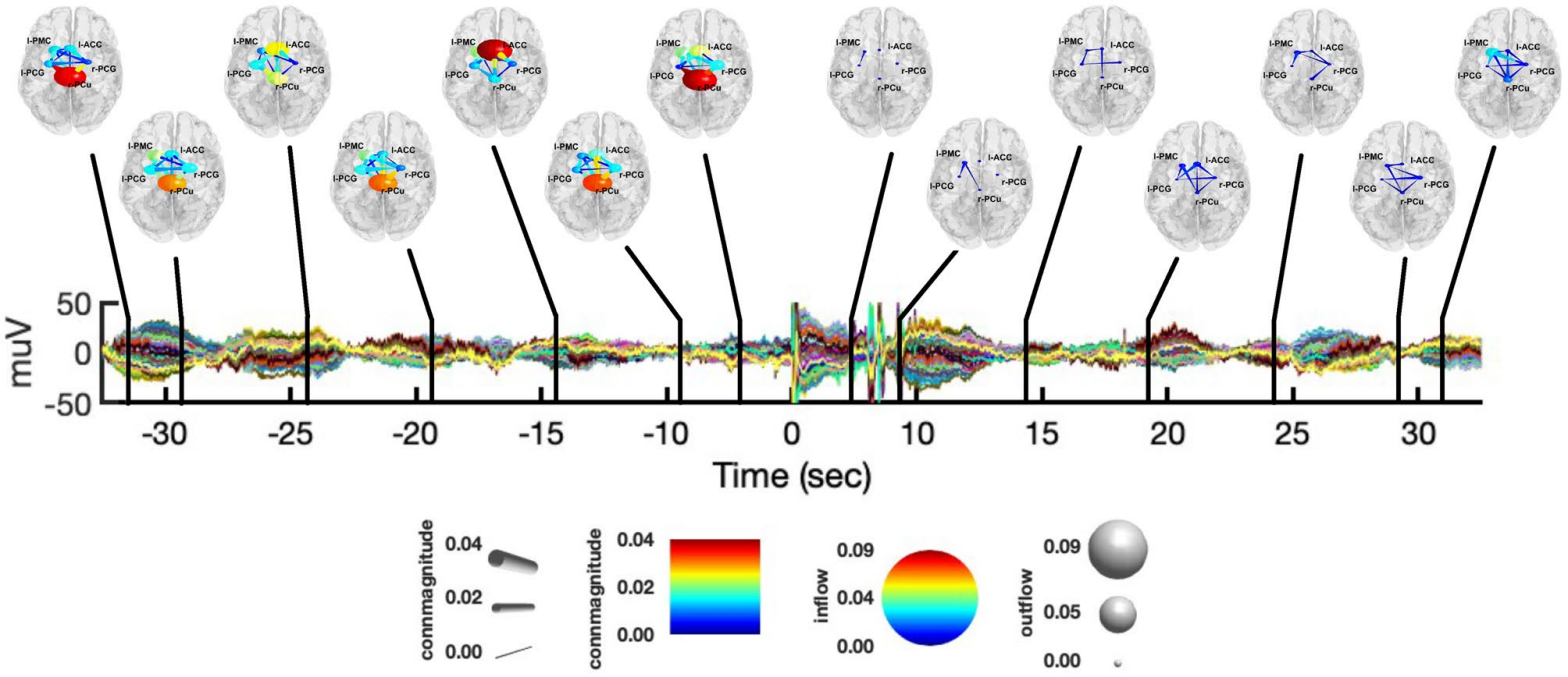
Graphical representation of causal interactions between the network nodes (i.e., l-PCG, l-PMC, r-PCu, l-ACC and r-PCG) integrated in the $\alpha \text{ and }\sigma$ bands, during both the resting-state from − 35 to − 5 s and CPT from + 5 to + 35 s, with respect to the CPT start. Nodes are reported in a standard template, with their corresponding label. Node size represent information outflow, whereas node color represents information inflow. The edges represent connections between nodes, whose color and size encode connectivity magnitude. The directionality of connections is given by the direction were the edges taper.

**Figure 8:**
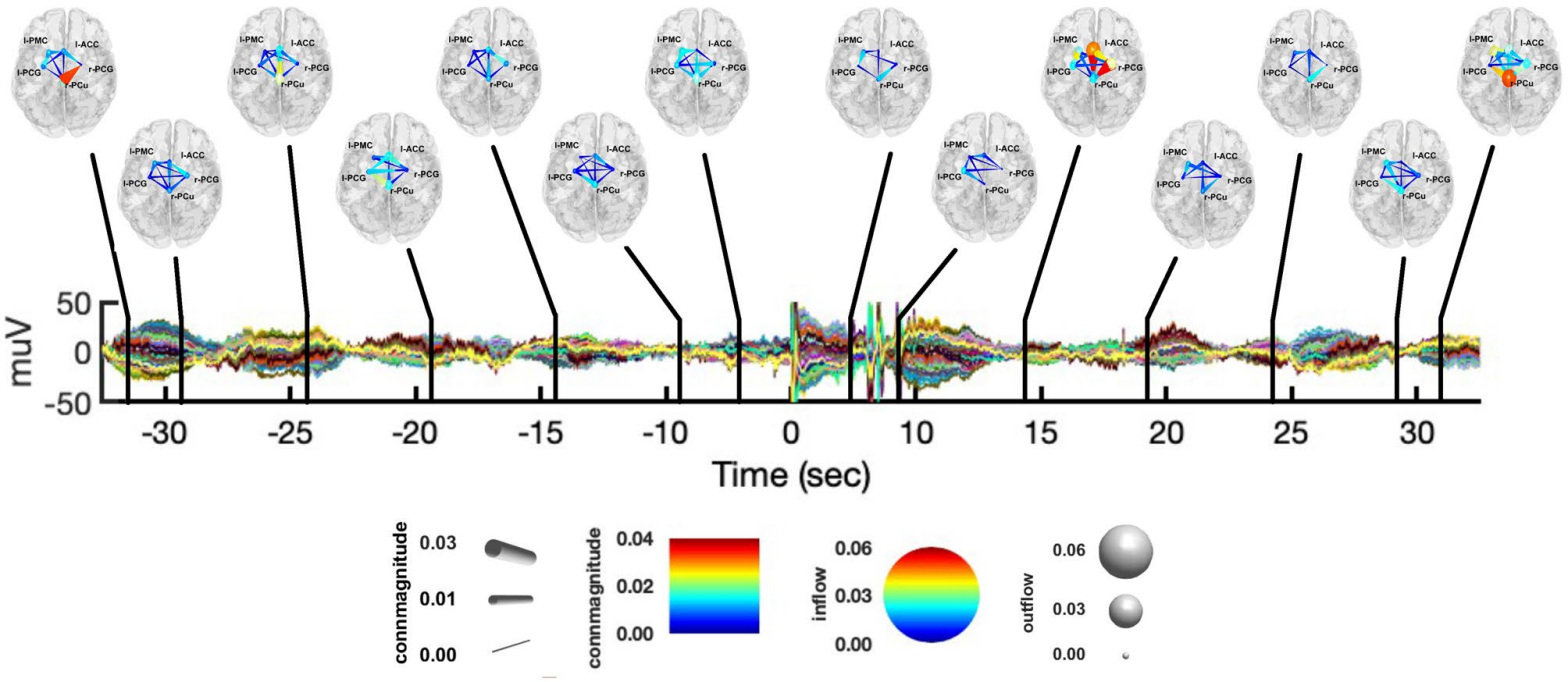
Graphical representation of causal interactions between the network nodes (i.e., l-PCG, l-PMC, r-PCu, l-ACC and r-PCG) integrated in the $\beta \; \text{and} \;\gamma$ bands, during both the resting-state from − 35 to − 5 s and CPT from + 5 to + 35 s, with respect to the CPT start. Nodes are reported in a standard template, with their corresponding label. Node size represent information outflow, whereas node color represents information inflow. The edges represent connections between nodes, whose color and size encode connectivity magnitude. The directionality of connections is given by the direction were the edges taper.

Figure 7 shows the interactions between nodes integrated in the $\alpha \text{ and } \sigma$ bands at different time instants, during both the resting-state and CPT. We chose to put together such frequency bands as they showed very similar patterns of connectivity. During the resting-state, we observed causal interactions involving all the network nodes, with the exception of r-PCG$\rightarrow$l-PCG. In particular, r-PCu and l-ACC were the nodes with the biggest outflow, whereas l-PCG, l-PMC and r-PCG were the nodes with the biggest inflow. Of note, this stable synchronization in the $\alpha \text{ and } \sigma$ bands was not present during CPT, with few or absent significant interactions between nodes. Finally, in Fig. 8, we report the causal interactions between nodes integrated in the $\beta \; \text{and} \;\gamma$ bands at different time instants, during both the resting-state and CPT. In such frequency range, we observed less specific connectivity patterns as compared to the other frequency bands, during both experimental conditions.

In what follows, we summarize the main findings presented above:

- from the K-means clustering of the IC’s scalp maps, we found a network of five group-common cortical nodes involved in the CPT task: the r-PCu, r-PCG, l-PCG, l-PMC, and l-ACC (Fig. 1).
- from the PSD analysis conducted at both the channel and source level (Figs. 2, 3), we observed an increase of power in the $\delta \; \text{and} \;\gamma$ bands during CPT, with respect to the resting-state. On the contrary, the $\alpha$-power decreased during CPT, with respect to the resting-state. We did not find any significant change in power for the $\theta ,\;\sigma ,\;{\text{and}}\;\beta$ bands.
- from the connectivity analysis (Fig. 4), we found $\delta$-band interactions characterizing the first windows of CPT, with l-PMC acting as the node with the greatest information outflow (Fig. 5). On the contrary, we did not find any specific $\delta$-band interaction during the resting-state. We found similar causal interactions in the $\theta$ band during CPT, whereas during the resting-state connectivity patterns were less specific (Fig. 6). We observed an opposite behavior for the $\alpha \;{\text{and}}\;\sigma$ bands, with a consistent flow of information among the network nodes during the resting-state. Particularly, the r-PCu and l-ACC were the nodes with the greatest outflow (Fig. 7). These patterns were almost totally suppressed from the start of CPT, with a partial recovery towards the end of the analyzed time interval. Finally, we did not observe any specific pattern of information flow for both the $\beta \;{\text{and}}\;\gamma$ frequency bands (Fig. 8).

## Discussion

In this work, we investigated the cortical changes associated with the early phase of the CPT through the analysis of both EEG power and connectivity estimates on a group of 18 healthy subjects. More specifically, we analyzed the dynamics of the PSD at both channel and source level during a CPT task, as well as the time-varying causal interactions occurring within a network of common ICs identified at the group level. Overall, our results suggest the presence of complex interactions occurring before and during the CPT that can be distinguished based on specific frequency bands, and which are accompanied by similar changes in power.

PSD analysis at both channel and source level revealed an increase of $\delta \;and\;\gamma \;{\text{activity,}}\;{\text{and}}\;{\text{a}}\;{\text{suppression}}\;{\text{of}}\;\alpha$ activity during the transition from rest to CPT, in line with previous work^27–31^. At the source level, significant changes were found in particular within l-ACC and r-PCG in the $\delta$ band, l-ACC, r-PCG and l-PMC in the $\gamma$ band, and r-PCG in the $\alpha \;{\text{band}}.\;{\text{Interestingly}},\;\delta \;{\text{and}}\;\gamma$ increases remained relatively stable within the analyzed CPT time-window (i.e., 5–35 s), whereas $\alpha$ changes reached statistical significance around 15–20 s after CPT onset.

Previous investigations did not provide a clear account regarding the physiological origin and functional meaning of EEG changes during CPT. The suppression of $\alpha \;{\text{and}}\;{\text{the}}\;{\text{increase}}\;{\text{of}}\;\gamma$ activity, especially within somatomotor areas contralateral to the stimulated hand have been interpreted as indicating regional activation related to stimulus processing^52^ and/or a shift in attention toward the stimulus rather than pain processing per se^27,53^. On another note, while $\delta \;{\text{activity}}\;{\text{is}}\;{\text{commonly}}\;{\text{interpreted}}\;{\text{as}}\;{\text{a}}\;{\text{sign}}\;{\text{of}}\;{\text{drowsiness}}\;{\text{and}}\;{\text{transition}}\;{\text{towards}}\;{\text{sleep,}}\;\delta$ waves or oscillations have been found in association with sudden increases in arousal and attention in response to external stimuli^54–57^. Indeed, during sleep, external stimuli can evoke a large positive-negative-positive waveform with a frontal maximum -the K-complex-^54^, whose generation and synchronization is thought to depend on the activation of subcortical, arousal-related structures with diffuse subcortico-cortical projections^58^. Similarly, during wakefulness, salient, arousing stimuli may induce evoked responses with a clear low-frequency component^55–57^. In addition, $\alpha$ activity is commonly higher during relaxed wakefulness and decreases during aroused states^59^. Given these considerations, the observed suppression of $\alpha \text{ and rise of }\delta$ activity could reflect a non-pain-specific arousal and attention-related response linked to stimulus’ salience.

The results of the connectivity analysis agreed with the observed changes in power, and revealed how the regions activated during the early phase of the CPT communicate with each other. Indeed, we found in the $\alpha$ band a suppression of interactions at the beginning of the CPT, which was followed by a partial recovery of connectivity toward the end of the examined time-window. The main connectivity hub for the $\alpha$ band was found in posterior areas, and in particular the r-Pcu. On the other hand, interactions in the $\delta$ band were almost absent before the beginning of the CPT, and increased in intensity and number during the first 10s of the task, especially around l-ACC and r-PCG regions. Afterwards, the interactions tended to become progressively weaker and less numerous. Overall, these results are consistent with a coordinated shift in connectivity patterns, from one dominated by $\alpha \text{ interactions during relaxed wakefulness with the eyes closed to one temporarily dominated by }\delta$ interactions immediately after the beginning of the CPT. The tendency of connectivity in these bands to return to the pre-CPT state toward the end of the examined time-window might indicate a partial habituation and a reduction of the arousing effects of the stimulus.

Of note, we did not observe any specific pattern of connectivity in the $\gamma$ band, although differences in power were consistent along the whole CPT. This could be related to the “short-range” nature of $\gamma$ connectivity that could be potentially not taken into account by our network. Indeed, the regions that contributed to the network were widespread among several distant brain regions. In this view, future analyses may consider different approaches to network identification. Here, we used a K-means algorithm with an optimal number of clusters as given by the Davis-Bouldin index. However, other hypothesis-driven approaches such as the *a priori* selection of brain regions could be adopted.

We restrained both the power and connectivity analysis to the first 30 s of the CPT task. The choice of such a window was motivated by previous analyses on these data^10^, in which an increase in EDASymp index is observed after the CPT onset with a peak after $\sim$30s from the stimulus. The EDASymp corresponds to the PSD of the EDA signal estimated in the 0.045-0.25 Hz frequency range and is a reliable index of sympathetic activity^11^. This is in line with previous studies reporting an increase of skin sympathetic activity in the median nerve occurring immediately after immersion of the hand in cold water^60^. Of note, early skin sympathetic activity is responsive to noxious or arousal stimuli, compared to other sympathetic correlates such as MSNA, which does not increase and sometimes tends to decrease in response to the arousal of the stimuli^17^. In this light, we reasonably assumed that the window used in our analysis was sufficiently long to investigate the cortical responses to the early sympathetic response elicited by CPT.

We did not consider the time interval ranging from the last 5sec of the initial resting-state condition to the first 5sec after the beginning of the CPT. Indeed, the physical interference induced by the experimenter guiding the volunteer’s hand into the water could not be neglected. Furthermore, the immediate cold sensation induced by the CPT at the beginning of the task dramatically affected the quality of EEG data by causing strong muscular artifacts. Accordingly, we attempted at minimizing the influence of such artifacts on the analysis. However, future studies could consider also other strategies to handle this aspect, as for instance using a different stimulation protocol or by employing other procedures for artifact correction.

Another limitation that should be considered is related to the estimation of cortical equivalent current dipoles following the clustering of single-subject ICA scalp maps. Indeed, we cannot exclude that the adoption of a standard template (i.e., the MNI template) for each subject may introduce approximations to the solution of the EEG inverse problem^61,62^. Accordingly, future studies may consider the integration of EEG measures with subject-specific head models as those obtained with MRI for enhancing the reliability of the labeled brain areas.

## Data Availability

The datasets used and analysed during the current study are available from the corresponding author on reasonable request.

## References

[1] Jänig, W. Autonomic nervous system. In *Human physiology*, 333–370 (Springer, 1989).

[2] Quadt L, Critchley H, Nagai Y (2022). Cognition, emotion, and the central autonomic network. Auton. Neurosci..

[3] Sklerov M, Dayan E, Browner N (2019). Functional neuroimaging of the central autonomic network: Recent developments and clinical implications. Clin. Auton. Res..

[4] Benarroch EE (1993). The central autonomic network: Functional organization, dysfunction, and perspective. Mayo Clinic Proceedings.

[5] Cechetto DF, Shoemaker JK (2009). Functional neuroanatomy of autonomic regulation. Neuroimage.

[6] Callara AL, Sebastiani L, Vanello N, Scilingo EP, Greco A (2021). Parasympathetic-sympathetic causal interactions assessed by time-varying multivariate autoregressive modeling of electrodermal activity and heart-rate-variability. IEEE Trans. Biomed. Eng..

[7] Fagius J, Karhuvaara S, Sundlof G (1989). The cold pressor test: Effects on sympathetic nerve activity in human muscle and skin nerve fascicles. Acta Physiol. Scand..

[8] Seals D (1990). Sympathetic activation during the cold pressor test: Influence of stimulus area. Clin. Physiol..

[9] Roatta S (1998). Effect of generalised sympathetic activation by cold pressor test on cerebral haemodynamics in healthy humans. J. Auton. Nerv. Syst..

[10] Ghiasi S, Greco A, Barbieri R, Scilingo EP, Valenza G (2020). Assessing autonomic function from electrodermal activity and heart rate variability during cold-pressor test and emotional challenge. Sci. Rep..

[11] Posada-Quintero HF (2016). Power spectral density analysis of electrodermal activity for sympathetic function assessment. Ann. Biomed. Eng..

[12] McGinley JJ, Friedman BH (2015). Autonomic responses to lateralized cold pressor and facial cooling tasks. Psychophysiology.

[13] Singh P, Khurana I (1991). Cardiovascular responses to cold pressor test: a test for autonomic functions. J. Indian Med. Assoc..

[14] Peng R-C (2015). Time-frequency analysis of heart rate variability during the cold pressor test using a time-varying autoregressive model. Physiol. Meas..

[15] Elias SO, Ajayi RE (2019). Effect of sympathetic autonomic stress from the cold pressor test on left ventricular function in young healthy adults. Physiol. Rep..

[16] Cui J, Wilson TE, Crandall CG (2002). Baroreflex modulation of muscle sympathetic nerve activity during cold pressor test in humans. Am. J. Physiol. Heart Circ. Physiol..

[17] Victor RG, Leimbach WN, Seals DR, Wallin BG, Mark AL (1987). Effects of the cold pressor test on muscle sympathetic nerve activity in humans. Hypertension.

[18] Hendriks-Balk MC (2020). Brainstem correlates of a cold pressor test measured by ultra-high field fmri. Front. Neurosci..

[19] Harper RM, Bandler R, Spriggs D, Alger JR (2000). Lateralized and widespread brain activation during transient blood pressure elevation revealed by magnetic resonance imaging. J. Comp. Neurol..

[20] Macey PM, Ogren JA, Kumar R, Harper RM (2016). Functional imaging of autonomic regulation: Methods and key findings. Front. Neurosci..

[21] Harper RM (2003). fMRI responses to cold pressor challenges in control and obstructive sleep apnea subjects. J. Appl. Physiol..

[22] La Cesa S (2014). fmri pain activation in the periaqueductal gray in healthy volunteers during the cold pressor test. Magn. Reson. Imaging.

[23] Lapotka M, Ruz M, Salamanca Ballesteros A, Ocón Hernández O (2017). Cold pressor gel test: A safe alternative to the cold pressor test in fMRI. Magn. Reson. Med..

[24] Frankenstein U, Richter W, McIntyre M, Remy F (2001). Distraction modulates anterior cingulate gyrus activations during the cold pressor test. Neuroimage.

[25] Richardson HL (2013). Neural and physiological responses to a cold pressor challenge in healthy adolescents. J. Neurosci. Res..

[26] Friston KJ (2011). Functional and effective connectivity: A review. Brain Connect..

[27] Chang PF, Arendt-Nielsen L, Chen AC (2002). Dynamic changes and spatial correlation of EEG activities during cold pressor test in man. Brain Res. Bull..

[28] Gram M, Graversen C, Olesen S, Drewes A (2015). Dynamic spectral indices of the electroencephalogram provide new insights into tonic pain. Clin. Neurophysiol..

[29] Ferracuti S, Seri S, Mattia D, Cruccu G (1994). Quantitative EEG modifications during the cold water pressor test: Hemispheric and hand differences. Int. J. Psychophysiol..

[30] Shao S, Shen K, Yu K, Wilder-Smith EP, Li X (2012). Frequency-domain EEG source analysis for acute tonic cold pain perception. Clin. Neurophysiol..

[31] Hansen TM (2017). Characterization of cortical source generators based on electroencephalography during tonic pain. J. Pain Res..

[32] Modares-Haghighi P, Boostani R, Nami M, Sanei S (2021). Quantification of pain severity using EEG-based functional connectivity. Biomed. Signal Process. Control.

[33] Korzeniewska A, Mańczak M, Kamiński M, Blinowska KJ, Kasicki S (2003). Determination of information flow direction among brain structures by a modified directed transfer function (ddtf) method. J. Neurosci. Methods.

[34] Gilbody S, Richards D, Brealey S, Hewitt C (2007). Screening for depression in medical settings with the patient health questionnaire (phq): A diagnostic meta-analysis. J. Gen. Intern. Med..

[35] Delorme A, Makeig S (2004). Eeglab: An open source toolbox for analysis of single-trial eeg dynamics including independent component analysis. J. Neurosci. Methods.

[36] Oostenveld, R., Fries, P., Maris, E. & Schoffelen, J.-M. Fieldtrip: open source software for advanced analysis of MEG, EEG, and invasive electrophysiological data. *Comput. Intell. Neurosci.* **2011** (2011). 10.1155/2011/156869 PMC3021840 21253357

[37] Winkler, I., Debener, S., Müller, K.-R. & Tangermann, M. On the influence of high-pass filtering on ica-based artifact reduction in eeg-erp. In *2015 37th Annual International Conference of the IEEE Engineering in Medicine and Biology Society (EMBC)*, 4101–4105 (IEEE, 2015). 10.1109/EMBC.2015.7319296 26737196

[38] Artoni F, Delorme A, Makeig S (2018). Applying dimension reduction to EEG data by principal component analysis reduces the quality of its subsequent independent component decomposition. Neuroimage.

[39] Mullen TR (2015). Real-time neuroimaging and cognitive monitoring using wearable dry EEG. IEEE Trans. Biomed. Eng..

[40] Chang C-Y, Hsu S-H, Pion-Tonachini L, Jung T-P (2019). Evaluation of artifact subspace reconstruction for automatic artifact components removal in multi-channel EEG recordings. IEEE Trans. Biomed. Eng..

[41] Billeci L (2023). A randomized controlled trial into the effects of probiotics on electroencephalography in preschoolers with autism. Autism.

[42] Palmer, J. A., Kreutz-Delgado, K. & Makeig, S. *Amica: An adaptive mixture of independent component analyzers with shared components* (Swartz Center for Computatonal Neursoscience, University of California San Diego, Tech. Rep, 2012).

[43] Delorme A, Palmer J, Onton J, Oostenveld R, Makeig S (2012). Independent EEG sources are dipolar. PLoS ONE.

[44] Pion-Tonachini L, Kreutz-Delgado K, Makeig S (2019). Iclabel: An automated electroencephalographic independent component classifier, dataset, and website. Neuroimage.

[45] Davies, D. L. & Bouldin, D. W. A cluster separation measure. *IEEE Trans. Pattern Anal. Mach. Intelli.* 224–227 (1979). 21868852

[46] Callara AL (2020). Ld-EEG effective brain connectivity in patients with cheyne-stokes respiration. IEEE Trans. Neural Syst. Rehabil. Eng..

[47] Lütkepohl H (2005). New Introduction to Multiple Time Series Analysis.

[48] Courellis H, Mullen T, Poizner H, Cauwenberghs G, Iversen JR (2017). EEG-based quantification of cortical current density and dynamic causal connectivity generalized across subjects performing bci-monitored cognitive tasks. Front. Neurosci..

[49] Pernet CR, Latinus M, Nichols T, Rousselet G (2015). Cluster-based computational methods for mass univariate analyses of event-related brain potentials/fields: A simulation study. J. Neurosci. Methods.

[50] Benjamini, Y. & Yekutieli, D. The control of the false discovery rate in multiple testing under dependency. *Ann. Stat.* 1165–1188 (2001).

[51] Theiler J, Eubank S, Longtin A, Galdrikian B, Farmer JD (1992). Testing for nonlinearity in time series: The method of surrogate data. Phys. D.

[52] Jensen O, Mazaheri A (2010). Shaping functional architecture by oscillatory alpha activity: Gating by inhibition. Front. Hum. Neurosci..

[53] Iannetti GD, Hughes NP, Lee MC, Mouraux A (2008). Determinants of laser-evoked EEG responses: Pain perception or stimulus saliency?. J. Neurophysiol..

[54] Colrain IM (2005). The k-complex: A 7-decade history. Sleep.

[55] Moayedi M (2015). Laser-evoked vertex potentials predict defensive motor actions. Cereb. Cortex.

[56] Wang, H. *et al.* Neural processes responsible for the translation of sustained nociceptive inputs into subjective pain experience. *Cereb. Cortex* (2022). 10.1093/cercor/bhac090 PMC9890464 35244170

[57] Brunia, C. H. M., van Boxtel, G. J. M. & Böcker, K. B. E. Negative slow waves as indices of anticipation: The bereitschaftspotential, the contingent negative variation, and the stimulus-preceding negativity. In *The Oxford Handbook of Event-Related Potential Components* (Oxford University Press, 2011).

[58] Siclari F (2014). Two distinct synchronization processes in the transition to sleep: A high-density electroencephalographic study. Sleep.

[59] Barry RJ, Clarke AR, Johnstone SJ, Magee CA, Rushby JA (2007). EEG differences between eyes-closed and eyes-open resting conditions. Clin. Neurophysiol..

[60] Fagius J, Blumberg H (1985). Sympathetic outflow to the hand in patients with Raynaud’s phenomenon. Cardiovasc. Res..

[61] Akalin Acar Z, Makeig S (2013). Effects of forward model errors on EEG source localization. Brain Topogr..

[62] Zanow F, Peters M (1995). Individually shaped volume conductor models of the head in EEG source localisation. Med. Biol. Eng. Comput..

