# Author Correction: EEG cortical activity and connectivity correlates of early sympathetic response during cold pressor test

**DOI:** 10.1038/s41598-023-30271-1

**Published:** 2023-02-22

**Authors:** Gianluca Rho, Alejandro Luis Callara, Giulio Bernardi, Enzo Pasquale Scilingo, Alberto Greco

**Affiliations:** 1 Dipartimento di Ingegneria dell’Informazione, University of Pisa, Via G. Caruso 16, 56122 Pisa, Italy; 2 Research Center “E. Piaggio”, University of Pisa, Largo Lucio Lazzarino 1, 56122 Pisa, Italy; 3 MoMiLab Research Unit, IMT School for Advanced Studies Lucca, 55100 Lucca, Italy

Correction to: *Scientific Reports* 10.1038/s41598-023-27480-z, published online 24 January 2023

The original version of this Article contained an error in the Acknowledgment section, where the project number for “Advanced technologies, methods, materials and heath analytics” was incorrectly given as “CUP: B83C22003920001” instead of “CUP: I53C22000780001”.

“The research leading to these results has received partial funding from the Italian Ministry of Education and Research (MIUR) in the framework of the CrossLab project (Departments of Excellence). This research has received partial funding from European Union Horizon 2020 Programme under grant agreement n 824153 of the project “POTION: Promoting Social Interaction through Emotional Body Odours”. Research partly funded by PNRR - M4C2 - Investimento 1.3, Partenariato Esteso PE00000013 - “FAIR - Future Artificial Intelligence Research” - Spoke 1 “Human-centered AI”, funded by the European Commission under the NextGeneration EU programme. This publication was produced with the co-funding European Union - Next Generation EU, in the context of The National Recovery and Resilience Plan, Investment 1.5 Ecosystems of Innovation, Project Tuscany Health Ecosystem (THE), Spoke 3 “Advanced technologies, methods, materials and heath analytics” CUP: B83C22003920001.”

now reads:

“The research leading to these results has received partial funding from the Italian Ministry of Education and Research (MIUR) in the framework of the CrossLab project (Departments of Excellence). This research has received partial funding from European Union Horizon 2020 Programme under grant agreement n 824153 of the project “POTION: Promoting Social Interaction through Emotional Body Odours”. Research partly funded by PNRR - M4C2 - Investimento 1.3, Partenariato Esteso PE00000013 - “FAIR - Future Artificial Intelligence Research” - Spoke 1 “Human-centered AI”, funded by the European Commission under the NextGeneration EU programme. This publication was produced with the co-funding European Union - Next Generation EU, in the context of The National Recovery and Resilience Plan, Investment 1.5 Ecosystems of Innovation, Project Tuscany Health Ecosystem (THE), Spoke 3 “Advanced technologies, methods, materials and heath analytics” CUP: I53C22000780001”.

The original Article has been corrected.

